# Le lupus érythémateux chronique: une nouvelle étiologie des macrochéilites

**DOI:** 10.11604/pamj.2013.16.142.3616

**Published:** 2013-12-18

**Authors:** Maha Mael-ainin, Karima Senouci

**Affiliations:** 1 Service de Dermatologie, CHU Ibn Sina, Université Med V, Souissi, Rabat, Maroc

**Keywords:** Macrochéilite, lupus érythémateux chronique, macrocheilia, chronic lupus erythematosus

## Images in medicine

Les macrochéilites sont rares et relèvent d'étiologies diverses dominées par les pathologies granulomateuses, notamment le syndrome de Melkersson Rosenthal, la maladie de Crohn ou la sarcoïdose. La macrochéilite au cours de la maladie lupique n'a jamais été décrite dans la littérature. En effet, l'atteinte labiale au cours du lupus chronique prend l'aspect de lésions d'abord érythémateuses puis blanchâtres situés au niveau du vermillon formant parfois un réseau lichénien et pouvant s'associer à des lésions érosives. Le lupus systémique se manifeste par des chéilites érosives et croûteuses gênant parfois l'alimentation. Ces lésions peuvent être inaugurales ou survenir lors d'une poussée. Le diagnostic étiologique des macrochéilites repose sur un faisceau d'arguments cliniques, paracliniques et évolutifs. La prise en charge des macrochéilites est difficile et non codifiée, diverses thérapeutiques ont été utilisées avec des résultats variables, notamment les corticoïdes par voie orale ou intralésionnelle, les antipaludéens de synthèse, les immunosuppresseurs, certains antibiotiques et thérapies ciblées, voir même le recours dans certains cas à la chéiloplastie de réduction. Nous rapportons le cas d'une patiente âgée de 30ans, suivie depuis 2003 pour la prise en charge d'un lupus érythémateux chronique. En 2010, la patiente a présenté une chéilite douloureuse de la lèvre supérieure augmentant progressivement de volume. L'examen clinique avait montré une macrochéilite squameuse de la lèvre supérieure, le reste de la muqueuse buccale était sans anomalie. Il n'y avait pas de paralysie faciale. L'examen de la muqueuse anale était normal. La biopsie labiale a montré une hyperkératose dyskératosique, des bouchons cornés, une hyperplasie pseudo-épithéliomateuse, un infiltrat inflammatoire du chorion sans granulome, l'immunofluorescence directe était négative, les anticorps anti-nucléaires étaient positifs à 1/160, les anticorps anti-ADN natif étaient négatifs. Le bilan à la recherche d'une localisation systémique de la maladie lupique était sans particularité. Le diagnostic du lupus érythémateux chronique a été retenu. La patiente a été mise sous Chloroquine à la dose de 4mg/kg/jour permettant la régression totale de la macrochéilite 6 mois après le début du traitement avec un recul de 2 ans.

**Figure 1:**
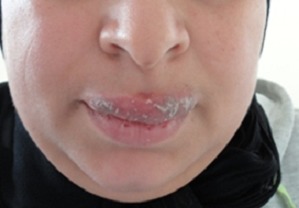
Macrochéilite squameuse de la lèvre supérieure

